# Knockout of the CMP–Sialic Acid Transporter SLC35A1 in Human Cell Lines Increases Transduction Efficiency of Adeno-Associated Virus 9: Implications for Gene Therapy Potency Assays

**DOI:** 10.3390/cells10051259

**Published:** 2021-05-19

**Authors:** Antje Banning, Anna Zakrzewicz, Xin Chen, Steven J. Gray, Ritva Tikkanen

**Affiliations:** 1 Institute of Biochemistry, Medical Faculty, University of Giessen, Friedrichstrasse 24, D-35392 Giessen, Germany; Antje.Banning@biochemie.med.uni-giessen.de (A.B.); Anna.Zakrzewicz@chiru.med.uni-giessen.de (A.Z.); 2 Department of Pediatrics, UTSW Medical Center, Dallas, TX 75390, USA; Xin.Chen@UTSouthwestern.edu (X.C.); Steven.Gray@UTSouthwestern.edu (S.J.G.)

**Keywords:** human gene therapy, AAV, sialic acid, sialylation, SLC35A1, glycosylation, aspartylglucosaminuria, neuronal ceroid lipofuscinosis

## Abstract

Recombinant adeno-associated viruses (AAV) have emerged as an important tool for gene therapy for human diseases. A prerequisite for clinical approval is an in vitro potency assay that can measure the transduction efficiency of each virus lot produced. The AAV serotypes are typical for gene therapy bind to different cell surface structures. The binding of AAV9 on the surface is mediated by terminal galactose residues present in the asparagine-linked carbohydrates in glycoproteins. However, such terminal galactose residues are rare in cultured cells. They are masked by sialic acid residues, which is an obstacle for the infection of many cell lines with AAV9 and the respective potency assays. The sialic acid residues can be removed by enzymatic digestion or chemical treatment. Still, such treatments are not practical for AAV9 potency assays since they may be difficult to standardize. In this study, we generated human cell lines (HEK293T and HeLa) that become permissive for AAV9 transduction after a knockout of the CMP–sialic acid transporter SLC35A1. Using the human aspartylglucosaminidase (*AGA*) gene, we show that these cell lines can be used as a model system for establishing potency assays for AAV9-based gene therapy approaches for human diseases.

## 1. Introduction

In the last years, an increased interest in generating recombinant adeno-associated virus (AAV)-based gene therapies for various human diseases has emerged. AAVs are DNA viruses of the Parvoviridae family, and they are non-pathogenic to humans. The AAV serotypes show different tissue tropisms due to using different cell surface structures as receptors. Thus, depending on the target tissue of the gene therapy approach, a suitable AAV serotype is selected that is capable of infecting the target organs. AAVs typically interact with a cell surface glycans, along with a co-receptor, to mediate cell surface binding and entry [1,2]. Cell surface glycans that contain terminal sialic acid residues function as receptors for some AAV serotypes. It has been shown that AAV serotypes 1, 4, 5 and 6 can bind sialic acid [3,4], and AAV2, 3 and 6 bind to heparin sulfate proteoglycans [5,6]. Other studies have also shown that heparan sulfate plays a role in the transduction of both AAV3 and AAV6. AAV2 and AAV3 transduction can be inhibited by adding heparin in cell culture [7]. However, AAV6 transduction is not inhibited by adding heparin in vitro [8], although the capsid does bind to heparin and can be purified using heparin columns. On the other hand, AAV9 binding to cell surface requires terminal galactose residues, especially in the Asn-linked glycans, and is inhibited by terminal sialic acid residues [9,10].

**Figure 1:**
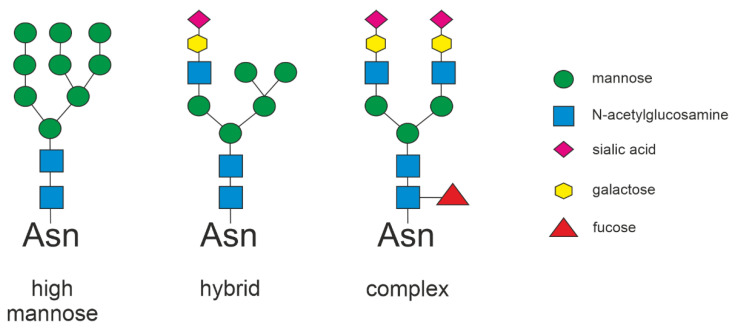
Three major types of N-glycans. N-glycans can be classified as high mannose, hybrid, or complex type. Each N-glycan comprises a core of N-acetylglucosamines and mannose. Sialic acid is commonly found as the terminal residue on complex N-glycans.

AAV9 is one of the most favored vectors for gene therapy approaches, in particular those targeting the brain, heart, and muscle [11]. This is due to its capability to infect many peripheral organs upon systemic administration. Furthermore, the propensity of AAV9 to transfer through the blood–brain barrier (BBB) after intravenous injection is an additional advantage, especially for treating diseases that affect the brain, such as lysosomal storage disorders [1,12,13,14,15]. In addition, intravenous gene therapy with an AAV9 vector, Onasemnogene abeparvovec, is currently also tested in clinical trials for various forms of spinal muscular atrophy (SMA) [16]. However, AAV9 poorly infects most cultured cells whose surface glycans tend to contain terminal sialic acid or heparin sulfate residues instead of galactose (Figure 1), which has made in vitro assays difficult to establish.

Extensive quality control of the virus lots intended for clinical trials is required before the vector can be used in humans. Thus, establishing an in vitro potency assay that can be carried out in cell culture and providing quantitative data on the transduction efficiency by each virus lot is a prerequisite for approval of AAV-based gene therapy products. The low in vitro transduction efficiency of AAV9 has largely prevented using common human cell lines that proliferate rapidly, such as HEK293T and HeLa cells. Although strategies for enhancing AAV9 transduction by removing the surface sialic acid have been suggested, e.g., by enzymatic removal of sialic acid or treatment with various chemicals (see discussion), such approaches are frequently inconvenient for practical work and difficult to standardize [9,10,17].

Aspartylglucosaminuria (AGU) is a lysosomal storage disorder caused by a deficiency of the *AGA* gene and the resulting lack of activity of the aspartylglucosaminidase (AGA) enzyme [18]. We have recently developed an AAV9-based gene therapy approach for AGU that was effective and safe in the *AGA* knockout mouse model [12]. To translate these findings into human AGU patients, it is necessary to develop an in vitro potency assay that can be used to assess the potency of the AAV lots intended for treating patients.

This study shows that knockout of the solute carrier family 35 member A1 (*SLC35A1*) gene encoding for the CMP–sialic acid transporter in human cell lines results in improved transduction efficiencies by AAV9, without affecting the cell viability. These cell lines show a significantly improved transduction over the parental cell lines. Thus, they are suitable for establishing potency assays for AAV9-mediated gene therapy vectors, especially after additionally knocking out the endogenous gene of interest.

## 2. Materials and Methods

### 2.1. Cell Culture

HEK293T (human embryonic kidney cells) and HeLa (human cervix carcinoma cells) cells were cultured in Dulbecco’s modified Eagle’s medium (DMEM) with high glucose, 10% fetal bovine serum (FBS), 1% penicillin/streptomycin (all from Gibco, Thermo Fisher Scientific, Dreieich, Germany). *AGA* knockout HEK293T cells without endogenous AGA expression were created by CRISPR/Cas9 and have been described before [19]. All cells were grown at 8% CO_2_ and 37 °C.

### 2.2. CRISPR/Cas9 Mediated Knockout of SLC35A1

For knocking out the expression of the sialic acid transporter SLC35A1 in HEK293T wild-type, HEK293T-*AGA*-knockout [19], and HeLa wild-type cells, the guide RNAs (gRNAs) were designed with the E-Crisp design tool (DKFZ, Heidelberg, Germany) and cloned via BbsI into pSpCas9(BB)-2A-Puro (PX459, Addgene plasmid #48139, a kind gift of Feng Zhang) [20]. The gRNA sequences are shown in Table 1. Twenty-four hours before transfection, cells were seeded onto 6-well plates. For transfections, 2.5 µg genome editing plasmid was transfected using MACSfectin™ (Miltenyi Biotec, Bergisch-Gladbach, Germany) according to the manufacturer’s protocol. After 24 h, the cells received a culture medium containing puromycin (2 µg/mL) for 24 h to eliminate untransfected cells. Thereafter, the cells were counted and seeded into 96-well plates (1 cell/well). Wells containing single-cell clones were expanded and used for further analysis. For analysis, cells were lysed with the Phire™ animal tissue direct PCR kit (Thermo Fisher Scientific, Dreieich, Germany). Genomic DNA was PCR amplified. The knockout was confirmed by sequencing the PCR products (Microsynth Seqlab, Göttingen, Germany). Primer sequences are shown in Table 1. Lack of protein expression was confirmed by immunofluorescence staining for SLC35A1.

### 2.3. AAV9 Vector Preparation and Quantification

Both research-grade AAV9/CBh-GFP [21] (AAV9/GFP) and AAV9/CBh-AGA [12] (AAV9/AGA) vectors for preclinical testing were manufactured at the University of North Carolina Vector Core (UNC-VC). The established plasmid was packaged into a self-complementary (sc) AAV9 vector [21] that is 10–100 times more efficient at transduction compared to traditional single-stranded (ss) AAV9 vectors [22,23]. The final purified product was dialyzed in phosphate-buffered saline (PBS) with an additional 212 mM NaCl and 5% D-sorbitol. Viral titer was determined by qPCR and confirmed by silver staining [24].

### 2.4. AAV9 Transduction

The day before the virus transduction, 100,000 cells per well were plated in 1 mL of media in a 12-well plate. Virus stocks were stored at −80 °C at 1 × 10^10^ vg/µL in PBS (5% *w/v* D-sorbitol). Cells were transduced with AAV9 viruses encoding either GFP or codon-optimized AGA at a multiplicity of infection (MOI) ranging from 25,000 to 500,000 viral genomes per cell. The virus was added directly into the culture medium. 24 h post-infection, the cells were transferred for another 24 to 48 h into 6 well plates for protein lysates, RNA or DNA isolation or were seeded onto coverslips for fluorescence microscopy. For protein lysates, the cells were washed in PBS and harvested in lysis juice (PJK, Kleinblittersdorf, Germany). Protein content was measured according to Bradford. For RNA, the cells were lysed in peqGOLD TriFast™ (VWR, Darmstadt, Germany). For isolation of genomic DNA, the cells were processed with the DNeasy blood and tissue kit (Qiagen, Hilden, Germany). Coverslips were fixed in 4% PFA for 10 min and mounted in ROTI ^®^ Mount FluorCare DAPI mounting medium.

### 2.5. SLC35A1 Cloning and Transfection

The coding region of *SLC35A1* was PCR amplified from HEK293T cells and cloned in-frame using XbaI and BamHI digestion into pExpr-IBA103 (IBA, Göttingen, Germany) with a C-terminal Twin-Strep-tag. (For primer sequences, see Table 1.) All constructs were verified by sequencing (Seqlab, Göttingen, Germany). For transient transfections, the cells were seeded onto 12-well plates 24 h before transfection, and 500 ng of plasmid DNA was transfected using MACSfectin™ (Miltenyi Biotec, Bergisch-Gladbach, Germany) according to the manufacturer’s protocol. The following day, the cells received the virus and were harvested 48 h post-transduction in lysis juice (PJK, Kleinblittersdorf, Germany).

### 2.6. Quantitative Real-Time PCR

For qPCRs, 3 µg of total RNA was reverse transcribed with 150 fmol oligo(dT) primers and M-MuLV reverse transcriptase (NEB, Frankfurt am Main, Germany) in a total volume of 45 µL. Genomic DNA was isolated with DNeasy blood and tissue kit (Qiagen, Hilden, Germany). Real-time quantitative PCRs were performed using the CFX Connect real-time PCR detection system (Bio-Rad, Feldkirchen, Germany). The annealing temperature was 60 °C for all primers. The reactions were done in duplicate with 20 ng cDNA or 1 µL of genomic DNA in a total volume of 10 µL using iTaqTM Universal SYBR Green Supermix (Bio-Rad, Feldkirchen, Germany). PCR products were quantified with standard curves based on purified PCR products ranging from 1 × 10^2^ to 1 × 10^8^ copies/µL. For normalization, the reference genes *GAPDH* and *Ywhaz* were used for cDNA, and *Lamin B2* and *AGA* were used for genomic DNA, respectively. For primer sequences, see Table 1.

### 2.7. Quantification of GFP Fluorescence

GFP fluorescence was measured in cell lysates using Tecan Infinite M200 plate reader (excitation 485 nm, emission 530 nm). Fluorescence was normalized to protein content. The number of GFP-positive cells was assessed microscopically with a Zeiss LSM710 confocal laser scanning microscope (Carl Zeiss, Oberkochen, Germany) and quantified with ImageJ. At least 300 cells per sample were counted per experiment.

### 2.8. Enzyme Activity Measurements

AGA activity was measured fluorometrically as described [25]. Reaction mixtures consisted of 20 µL cell lysate (blank: lysis buffer) and 20 µL of Asp-AMC (L-Aspartic acid β-(7-amido-4-methylcoumarin); 50 µM in McIlvain’s phosphate-citrate buffer pH 6.5). Samples were incubated for 24 h at 37 °C, after which the reaction was terminated by adding 200 µL McIlvain’s buffer pH 4.5. A standard curve containing 0 to 1000 pmol of 7-amino-4-methylcoumarin (AMC, Sigma-Aldrich, Taufkirchen, Germany) was measured in parallel. The standard curve was supplemented with Asp-AMC to keep the total concentration of Asp-AMC and AMC constant. All samples were measured in triplicates with a Tecan Infinite M200 plate reader using 355 nm excitation and 450 nm emission wavelengths, respectively. AGA activity was expressed as µmol AMC/mg protein.

### 2.9. Antibodies

A rabbit antiserum against the α and β subunits of AGA was used to detect AGA in Western blots [26]. A mouse monoclonal antibody against GAPDH was purchased from Abcam (Cambridge, UK). For staining of SLC35A1, a polyclonal rabbit antibody was obtained from Proteintech (St. Leon-Rot, Germany), diluted 1:200 and used on methanol-fixed cells.

### 2.10. Lectin Staining

Cells were grown on coverslips and fixed with 4% PFA for 10 min. Surface carbohydrates were labeled with 20 µg/mL Cy3-coupled *Sambucus nigra* lectin (SNA) or fluorescein-coupled *Erythrina crista-galli* lectin (ECL) obtained from Vector Laboratories (Biozol, Eching, Germany) for 20 min at room temperature. After two washes with PBS to remove unbound lectins, samples were mounted with ROTI^®^Mount FluorCare DAPI (Carl Roth, Karlsruhe, Germany) and imaged with a Zeiss LSM710 confocal laser scanning microscope (Carl Zeiss, Oberkochen, Germany)

To detect cell surface galactose with a plate reader, living cells were incubated with 10 µg/mL fluorescein-coupled *Erythrina crista-galli* lectin (ECL) and 1 µg/mL DAPI in a 12 well-plate for 30 min in DMEM without serum at 37 °C. The cells were washed with PBS, trypsinized, and resuspended in 300 µL PBS. The cells were counted. The fluorescence was measured with Tecan Infinite M200 plate reader using excitation/emission of 494/521 nm for ECL and excitation/emission: 365/450 nm for DAPI. Non-stained cells were used as background control.

### 2.11. Assessment of Cell Viability

The proliferation of the parental HEK293T and Hela and the respective *SLC35A1* knockout cells was determined at 24 h intervals over 3 days of culture using an MTT [3-(4,5-dimethylthiazol-2-yl)-2,5-diphenyltetrazolium bromide] assay. For the assessment, the cells were seeded onto 96-well plates at a density of 2.5 × 10^3^ cells per well. On the day of the measurement, MTT (Sigma-Aldrich, Taufkirchen, Germany) was added to a final concentration of 0.5 mg/mL. After 40 min incubation, the medium was removed. The cells were lysed in 100 µL lysis buffer (containing 5% formic acid and 95% isopropanol) per well. Then, absorbance recorded using a microplate reader at a wavelength of 550 nm (with a reference wavelength of 690 nm). Six replicas for each time point were included in each experiment, and 5 independent experiments were performed. The average absorbance measured for the parental cells at 24 h was set as 1. The corresponding normalized values (relative proliferation) for the other data points were calculated.

### 2.12. Statistical Analysis

All experiments were performed at least three times. Data are expressed as mean ± SD. Statistical comparisons between groups were made using Student’s t-tests, 1way or 2way ANOVA, as appropriate (GraphPad Prism 5, San Diego, CA, USA). Values of *p* < 0.05 were considered significant (*), while values of *p* < 0.01 were considered very significant (**) and *p* < 0.001 extremely significant (***).

## 3. Results

**Figure 2:**
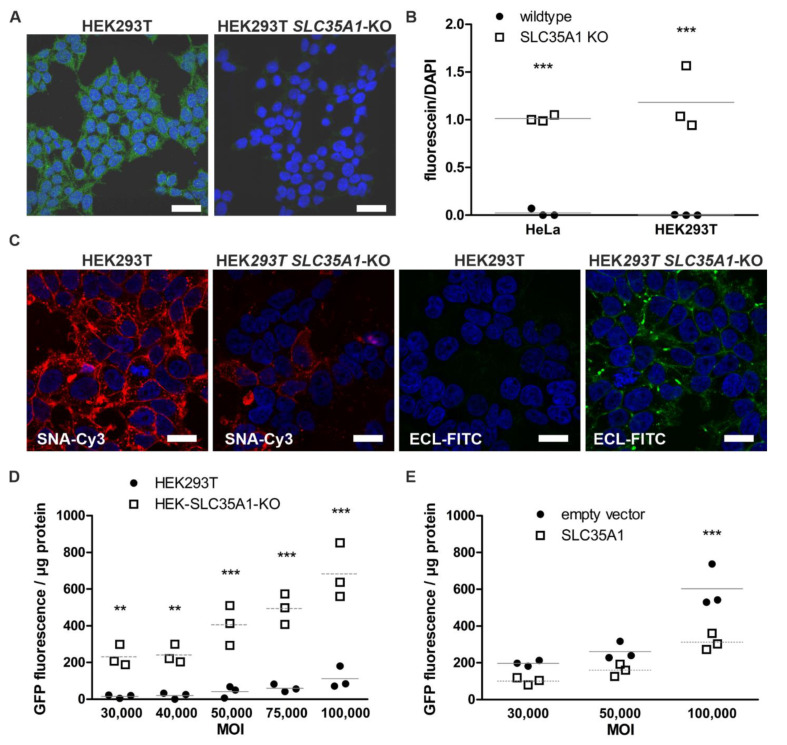
Knockout of the sialic acid transporter SLC35A1 in HEK293T cells improves the transduction efficiency of AAV9. (**A**) HEK293T wild-type and *SLC35A1* knockout cells were grown on coverslips and stained for SLC35A1. Anti-rabbit IgG coupled to Alexa Fluor^®^ 488 was used as a secondary antibody. In contrast to HEK293T wild-type cells, *SLC35A1* knockout cells lack any specific immunoreactivity signal for *SLC35A1*. Scale bar 20 µm. (**B**) Staining of *SLC35A1* knockout HEK293T cells with FITC-coupled *Erythrina crista-galli* lectin. The cells were incubated with the lectin (10 µg/mL, 30 min at 37 °C), and the fluorescence was measured using a plate reader. The results are shown for 4 independent experiments. (**C**) *SLC35A1* knockout cells show higher fluorescence after staining with ECL lectin and reduced fluorescence upon staining with SNA lectin. PFA-fixed cells were subjected to lectin staining using 20 µg/mL FITC-labeled ECL lectin, recognizing terminal galactose, or Cy-3-labeled SNA lectin, recognizing terminal sialic acid. Scale bar 10 µm. (**D**) Knockout of *SLC35A1* improves AAV9 transduction efficiency. Cells were infected with increasing amounts of AAV9/GFP, and the GFP fluorescence was measured in cell lysates 48 h post-transduction. (**E**) Transient SLC35A1 expression after plasmid transfection reduces AAV9/GFP transduction efficiency. Data in (**D**,**E**) represent 3 independent experiments. Statistics with 2way ANOVA against wild-type cells (**B**,**D**) or empty vector (**E**). ** *p* ≤ 0.01, *** *p* ≤ 0.001. Horizontal lines in (**B**,**D**,**E**) indicate the mean.

Transduction efficiency of cultured human cell lines with AAV9 serotype is generally very low due to the terminal sialic acid residues that obscure the galactose residues in the cell surface glycosylated structures needed by AAV9 for cell entry [9,10,27]. We thus identified potential targets for a gene knockout that might increase the AAV9 transduction efficiency. The CMP–sialic acid transporter (SLC35A1) is responsible for transferring CMP–sialic acid from the cytosol through Golgi membranes, thus playing a vital role in the terminal sialylation of glycan structures [28]. We knocked out the *SLC35A1* gene by CRISPR/Cas9 technology in HEK293T cells. We produced single-cell clones containing a genomic insertion of 1 bp (Supplementary Figure S1A), resulting in a frameshift and absence of CMP–sialic acid transporter expression by immunostaining (Figure 2A). Cell viability of the knockout cells was not impaired by the *SLC35A1* knockout, as evidenced by the cell proliferation assay (Supplementary Figure S1B). Staining with fluorescein isothiocyanate-coupled *Erythrina crista-galli* lectin that binds to terminal galactose and N-acetyl-galactosamine residues showed that, compared to the parental cell line, the *SLC35A1* knockout cells exhibit more free binding sites of the lectin (measured as total lectin fluorescence normalized to DAPI staining), as would be expected after loss of terminal sialic acid residues (Figure 2B). Fluorescent staining with Cy3-coupled *Sambucus nigra* lectin (SNA) that binds to sialic acid exhibited lower staining in *SLC35A1* knockout cells than in the parental HEK293T cells (Figure 2C). Staining with FITC-coupled *Erythrina crista-galli* lectin revealed brighter staining in *SLC35A1* knockout cells, consistent with the presence of more terminal galactose residues (Figure 2C).

The *SLC35A1* knockout clone and the parental HEK293T cells were transduced with different amounts of AAV9/GFP (MOI 3 × 10^4^ to 1 × 10^5^). The resulting GFP fluorescence was measured using a plate reader and normalized to protein amount in the lysates (Figure 2D). At all MOI used and in a dose-dependent fashion, *SLC35A1* knockout cells showed a significantly higher GFP fluorescence (average 10.5 times over the MOI range used) than the parental cells. Expression of the *SLC35A1* gene product CMP–sialic acid transporter by transient transfection of an expression plasmid decreased the AAV9/GFP transduction efficiency (Figure 2E), demonstrating that the improved transduction efficiency was due to the *SLC35A1* gene knockout.

For human gene therapy, potency assays that measure the functional activity of the gene therapy vector are essential. The use of cell line-based potency assays has greatly been hampered by the low transduction efficiency of human cell lines by some of the AAV serotypes typically used for gene therapy, in particular AAV9. To test the suitability of the *SLC35A1* knockout cells for a potency assay, we used an AAV9 vector (AAV9/AGA) that carries the optimized human *AGA* gene coding for aspartylglucosaminidase (AGA) that is deficient in aspartylglucosaminuria (AGU). We have recently shown in an AGU mouse model that this vector shows a high potential for clinical translation [12]. We thus generated double knockout of *AGA* and *SLC35A1* in HEK293T cells by knocking out the *SLC35A1* gene in our previously characterized *AGA* HEK293T knockout cells [19]. Cell clones 1 and 34 showed no expression of the CMP–sialic acid transporter (Supplementary Figure S2) and contained a 1 bp or 2 bp genomic insertion within the open reading frame of the *SLC35A1* gene (Supplementary Figure S3) were chosen for further studies. The two *AGA*/*SLC35A1* double knockout clones (clone 1 and 34) showed a significantly improved, dose-dependent transduction efficiency by AAV9/GFP than HEK293T cells (Supplementary Figure S4). Interestingly, at higher MOI, also the single *AGA* knockout cells were significantly better transduced by AAV9/GFP than the parental cell line. The reason for this enhanced transduction efficiency is not clear, but it may result from the impairment of lysosomal function due to *AGA* knockout. This is likely to result in altered degradation of glycosylated and thus also sialylated structures in lysosomes. This, in turn, may result in the downregulation of sialic acid-containing structures on the cell surface, enhancing AAV9 transduction. All further experiments were performed with the *AGA*/*SLC35A1* double knockout clone 1.

**Figure 3:**
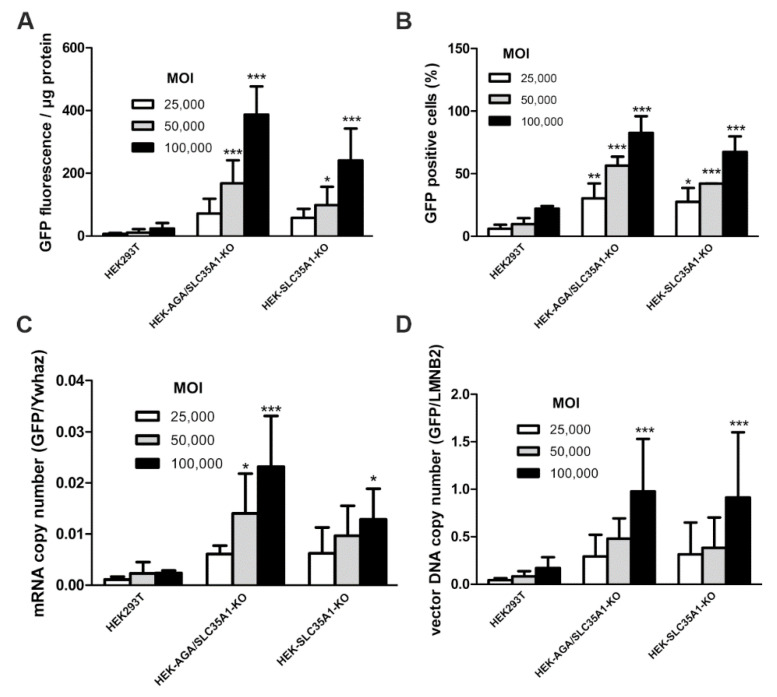
Comparison of AAV9/GFP transduction efficiency of HEK293T wild-type, *SLC35A1/AGA* double knockout and *SLC35A1* single knockout cells. Cells were infected with increasing amounts of AAV9/GFP. 72 h post-infection, the cells were harvested and (**A**) GFP fluorescence in cell lysates was quantified. (**B**) 24 h post-infection, cells were transferred onto coverslips and grown for another 24 h. The cells were fixed and analyzed by fluorescence microscopy. At least 300 cells per sample were counted per experiment. Only cells with a distinct green fluorescence signal were counted as GFP-positive. (**C**,**D**) Genomic DNA and mRNA were isolated from the cells. The copy number of the GFP mRNA (**C**) and vector DNA (**D**) were analyzed by quantitative real-time PCR. (**A**–**D**) Bars show means ± SD of 3 independent experiments, 2way ANOVA against HEK293T. * *p* ≤ 0.05, ** *p* ≤ 0.01, *** *p* ≤ 0.001.

Direct comparison of the *SLC35A1* single knockout and the *AGA*/*SLC35A1* double knockout showed that both cell lines were efficiently transduced by the AAV9/GFP vector, and both the GFP fluorescence (Figure 3A) and the number of GFP-positive cells (Figure 3B and Supplementary Figure S5) increased with an increasing MOI. The success of the viral transduction was also determined by qPCR by measuring the copy number of the GFP mRNA (Figure 3C, normalized to the mRNA of the endogenous *Ywhaz* gene) or vector DNA (Figure 3D, normalized to endogenous *LMNB2* gene). Supplementary Figure S6 shows the graphs from Figure 3A–D with all data points.

**Figure 4:**
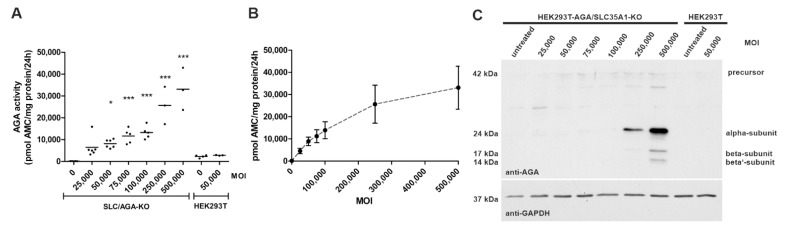
Infection of *AGA/SLC35A1* double knockout cells with AAV9/AGA virus results in AGA protein expression and enzyme activity. The cells were infected with increasing amounts of AAV9/AGA and harvested 72 h post-infection for analysis of AGA activity (**A**,**B**) or AGA expression by Western blot (**C**). (**A**,**B**) AGA activity was measured fluorometrically, statistical analysis by 1way ANOVA against HEK293T without virus transduction. Graphs show the data for 3 independent experiments. * *p* ≤ 0.05, *** *p* ≤ 0.001. Horizontal lines indicate the mean. (**C**) Cell lysates were analyzed by SDS–PAGE and Western blot for AGA expression. GAPDH was used as a loading control.

Transduction of the *AGA*/*SLC35A1* double knockout cells with increasing MOI (2.5 × 10^4^ to 5 × 10^5^) of the AAV9/AGA virus resulted in a significantly increased AGA enzyme activity in a nonlinear and saturable fashion (Figure 4A,B). Western blot analysis of the transduced cells using an anti-AGA antibody showed a detectable level of AGA protein expression at MOI 1 × 10^5^ and higher. Importantly, the AGA precursor polypeptide was processed into the α and β subunits (Figure 4C) required for the enzyme activity.

**Figure 5:**
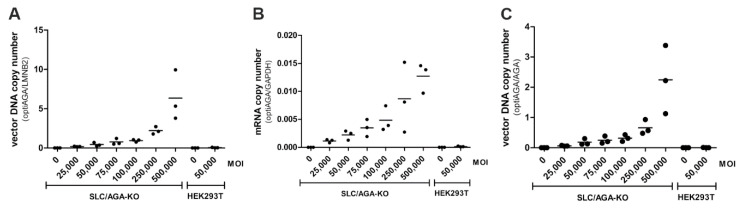
Transduction of *AGA/SLC35A1* double knockout HEK293T cells with AAV9/AGA virus: assessment of the transgene delivery. The cells were transduced with increasing amounts of AAV9/AGA. 72 h post-infection, the cells were harvested to analyze genomic DNA (**A**,**C**) or quantitative PCR of cDNA (**B**). The codon-optimized *AGA* genomic DNA or cDNA was amplified by quantitative PCR and normalized to Lamin B2 (*LMNB2*, (**A**)), *GAPDH* (**B**), or endogenous *AGA* (**C**). In cells without AAV9/AGA, no signal beyond background for codon-optimized *AGA* was found. Graphs show the data for 3 independent experiments. Horizontal lines indicate the mean.

Successful transduction was also shown by demonstrating an increased level of the codon-optimized *AGA* transgene at the vector DNA (Figure 5A, normalized to endogenous *LMNB2* gene) and mRNA level (Figure 5B, normalized to *GAPDH* mRNA). Compared to the glycolytic enzyme GAPDH, the mRNA level of the *AGA* transgene is very low (Figure 5B). However, a comparison of the optimized and the endogenous *AGA* gene showed that at the highest MOI, the viral genome is present in the *AGA*/*SLC35A1* double knockout cells at about two-fold excess to the endogenous *AGA* gene (Figure 5C). There is a minor difference in the vector DNA copy numbers when using endogenous *LMNB2* (about 6) and endogenous *AGA* (about 2) as a reference. This may be because HEK293T cells are partially polyploid, and the copy number of the reference chromosomes may be different. Taken together, these data show that knockout of *SLC35A1* in HEK293T cells results in significantly improved transduction and transgene expression mediated by AAV9-based vectors.

**Figure 6:**
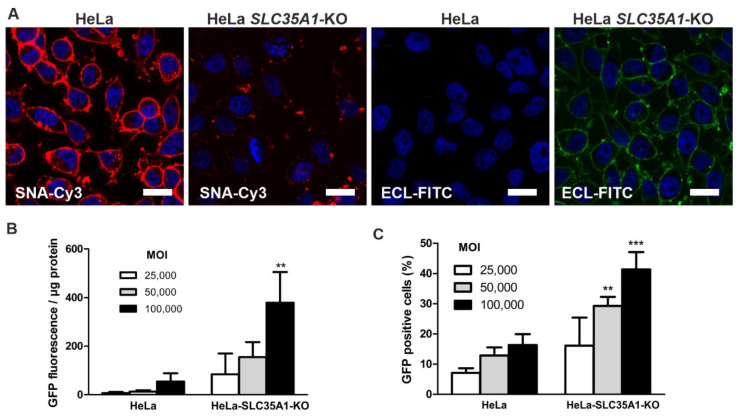
Knockout of *SLC35A1* in HeLa cells improves AAV9/GFP transduction efficiency. (**A**) *SLC35A1* knockout HeLa cells show higher fluorescence after staining with ECL lectin and reduced fluorescence upon staining with SNA lectin. Cells were fixed with PFA and stained with 20 µg/mL FITC-labeled ECL lectin, recognizing terminal galactose, or Cy-3-labeled SNA lectin, recognizing terminal sialic acid. Scale bar 10 µm. (**B**) The cells were infected with increasing amounts of AAV9/GFP, and the GFP fluorescence was measured in cell lysates 48 h post-transduction. (**C**) At 24 h post-infection, the cells were transferred onto coverslips and grown for a further 24 h. The cells were fixed and analyzed by fluorescence microscopy. At least 300 cells per sample were counted per experiment. Only cells with a distinct green fluorescence signal were counted as GFP-positive. Bars show means ± SD of 3 independent experiments, 2way ANOVA against HeLa. ** *p* ≤ 0.01, *** *p* ≤ 0.001.

To show that the *SLC35A1* gene knockout results in improved transduction efficiencies also in further human cell lines, we produced knockout HeLa cell clones that showed a 1 bp genomic insertion in the *SLC35A1* gene (Supplementary Figure S7). As with the *SLC35A1* knockout HEK293T cells, the viability was not compromised (Supplementary Figure S7). More terminal galactose residues and fewer sialic acid residues were present on the cell surface of the *SLC35A1* knockout HeLa cells (Figure 6A). Transduction of the *SLC35A1* knockout HeLa cells with the AAV9/GFP vector resulted in improved transduction compared with the parental HeLa cells (Figure 6B,C and Supplementary Figure S8). However, the percentage of GFP-expressing cells was somewhat lower in HeLa cells (Figure 6C) than in the HEK293T cells (Figure 3B). Supplementary Figure S9 shows the graphs in Figure 6B,C with all data points. These data show that knockdown of the *SLC35A1* gene efficiently increases the transduction efficiency of AAV9 in human cell lines.

## 4. Discussion

In previous studies, solutions to overcome the low infection efficiency of proliferating cells and cultured cell lines by AAV9 have been proposed. These include using the Chinese hamster ovarian (CHO) Lec2 mutant cell line that is unable to transport CMP–sialic acid to Golgi lumen [29,30], enzymatic removal of cell surface sialic acid determinants [9,10,11] and treatment of cells with various chemicals, such as compound C that inhibits AMP-dependent protein kinase (AMPK), or etoposide [17,31].

As compared to the parental CHO Pro5 cells, Lec2 cells can efficiently be infected with AAV9 [9,10]. However, Lec2 cells may be suboptimal for potency assays aiming at a functional assessment of the human transgene since post-translational modifications, processing and trafficking of the human protein may be altered in these cells. In addition, protein–protein interactions with non-human binding partners may be impaired, resulting in altered function. A further HEK293-based cell line has been described that exhibits modestly enhanced AAV9 transduction efficiencies [32]. The HEK293 2v6.11 cell line expresses the adenovirus E4 protein in an ecdysone-inducible manner [33]. It has been shown to be suitable for assays that address neutralizing antibodies against some AAV serotypes [32]. However, similar to other compound treatments, ecdysone induction adds another variable to the assays, limiting the wider use of this cell line.

The human cell lines developed in the present study are based on a genetic knockout of the CMP–sialic acid transporter and thus exhibit the same defect in sialylation as the Lec2 cells. Functional ablation of the CMP–sialic acid transporter by *SLC35A1* gene knockout provides means for increasing the infection efficiency by AAV9 in cell lines that divide rapidly. This is due to the lack of sialic acid residues in the glycan determinants at the cell surface, providing improved access of AAV9 to its main receptors, terminal galactose residues [9,10]. Our system is based on genome-edited cell lines. It would thus be advisable to control the cell lines intended for a potency assay by targeted deep sequencing and/or karyotyping to ensure that they remain genetically stable. However, we have not observed any changes in the phenotypes (e.g., AGA enzyme activity) in our *AGA* knockout cell lines originally published in 2016 that have since been used for further studies [19]. The double knockout cell lines used in this study were established already in 2018. Our long-term observations do not imply that genetic instability or loss of homogeneity would be a problem with these cell lines.

Enzymatic removal of sialic acid by neuraminidase is a method that does not require any genetic modification of the target cells and thus facilitates using various cell lines for AAV transduction [9,10,11]. It has been shown that intranasal, intravenous or intramuscular neuraminidase pretreatment to remove terminal sialic acid in vivo in mouse models can substantially enhance AAV9 transduction efficiency in specific tissues [9,10,11,34]. However, the results of a potency assay involving a neuraminidase treatment depend on the efficiency of the sialic acid removal. They may thus vary from assay to assay. Therefore, such an assay may be difficult to standardize and validate. In addition, the costs of the assay are increased due to the requirement for the recombinant enzyme. The *SLC35A1* knockout cell lines that we have generated in this study do not require any further modifications or treatments before transduction by AAV9, making assays that are based on our cell lines easier to standardize and validate.

Recently, Krotova and Aslanidi studied several AAV serotypes that carried the firefly luciferase (Luc) transgene [17]. The authors used a relatively low MOI (e.g., 2000 vg/cell for AAV9), which is probably due to the higher sensitivity of Luc activity measurement than the methods used in our study. They showed that inhibition of AMPK with 10 µM compound C (also known as dorsomorphin) results in a several-fold increase in Luc activity than non-treated, Luc-AAV-infected cells. Treatment with compound C, together with the cytokines interleukin 6 and tumor necrosis factor α, further increased the luciferase activity for most of the AAV serotypes tested (except AAV3). However, treatment with these three compounds also resulted in mild impairment of cell viability. Unfortunately, compound C is not specific for AMPK but also inhibits numerous other kinases [35]. Thus, it should be used with great caution in assays that aim at the functional measurement of the transgene. In addition, depending on the AAV serotype, the effect of compound C treatment on the transduction efficiency varied largely.

In addition to potency assays, the cell lines generated in the present study may also be useful for in vitro assays that assess the presence of neutralizing antibodies against AAV in patients that are to be treated with gene therapy. Since a very large fraction of humans, estimated to be about 90% of adults, has had contact with naturally occurring, non-pathogenic AAV variants early in life, there is a risk of the prior existence of antibodies against AAV in patients to be treated [36]. Not all of these antibodies are necessarily neutralizing. Still, an uncomplicated and fast in vitro assay assessing such antibodies would be of great use for gene therapy trials. This could be accomplished by incubating the viral vector, preferably AAV9/Luc, with patient sera and then testing the efficiency of the infection in the cell systems described in this study.

In this study, we have shown that knockout of the CMP–sialic acid transporter *SLC35A1* gene substantially increases the transduction efficiency of AAV9 in two human cell lines that are easy to culture. The *SLC35A1* gene knockout did not result in a decreased viability of these cell lines, despite the sialylation defect, and additional genes can be knocked out in these cell lines without affecting viability. Therefore, by knocking out a gene of interest, e.g., the target gene for a gene therapy approach, it is possible to generate cell lines that do not express the endogenous protein and only express the transgene delivered by the AAV9 vector. This facilitates establishing potency assays without an endogenous background activity. Likely, the AAV9 tropism towards cultured human cell lines can generally be improved using *SLC35A1* knockout, facilitating the generation of further cell lines beyond those tested in the present study that can be used for studies with AAV9.

Although our system may not be useful for viruses other than AAV9 that do not use the same receptor, it will certainly be of interest for studies using variants derived from AAV9, such as PHP.B and PHP.eB [37,38]. Although the identity of the cellular receptor of these variants is not quite clear, they contain the five amino acids that have been shown to mediate galactose binding in AAV9 and are thus also likely to bind to galactose [39]. Thus, it is plausible that the transduction efficiencies of these variants, which show good penetration of the blood–brain barrier and are thus of great interest for human gene therapy, can be improved by *SLC35A1* knockout in cultured cells.

## Figures and Tables

**Table 1:** Sequences of the primers. All primer sequences are given in the 5′–3′ direction.

Primer Name	Sequence 5′–3′
SLC35A1 gRNA fwd	CACCGGGTATAGACTGCAGCCATCA
SLC35A1 gRNA rev	AAACTGATGGCTGCAGTCTATACCC
SLC35A1 screening fwd	ACTAAGTAATGTCTTTGTTGCACG
SLC35A1 screening rev	TGTTTAGCAGCATCCTTGGTC
SLC35A1-Start-XbaI	CTATATCTAGAATGGCTGCCCCGAGAGACAAT
SLC35A1-nonstop-BamHI	CTATAGGATCCCACACCAATAACTCTCTCCTTTG
EGFP fwd	AGCAGCACGACTTCTTCAACTCC
EGFP rev	TGTAGTTGTACTCCAGCTTGTGCC
AGA-codon-optimized fwd	ACAGGACATCCCCATCCATA
AGA-codon-optimized rev	TCGGGCTATCTCCGACTCTA
GAPDH fwd	CATCTTCCAGGAGCGAGATCCC
GAPDH rev	CCAGCCTTCTCCATGGTGGT
Ywhaz fwd	AGGTTGCCGCTGGTGATGAC
Ywhaz rev	GGCCAGACCCAGTCTGATAGGA
Lamin B2 fwd	GTTAACAGTCAGGCGCATGGGCC
Lamin B2 rev	CCATCAGGGTCACCTCTGGTTCC
AGA-genomic fwd	TCCCTGACTTAACTGCCCTCGT
AGA-genomic rev	TGAATCGTATGCACTGCCACCA

## Data Availability

The data are available from the corresponding author upon a reasonable request.

## Supplementary Materials

The following are available online at https://www.mdpi.com/article/10.3390/cells10051259/s1, Figure S1: Genomic sequencing and cell viability of *SLC35A1* knockout HEK293T cells. (A) CRISPR/Cas9-mediated knockout of the *SLC35A1* gene was confirmed by sequencing of PCR-amplified genomic DNA surrounding the gRNA targe_ng site. Knockout cells show frameshi muta_ons due to an inser_on of 1 base 4 bp upstream of the Cas9 PAM sequence. (B) Cell viability of the parental and *SLC35A1* knockout cells was determined using the MTT assay and is given as rela_ve prolifera_on normalized to 24 h _me point of the parental cell line. The results represent the mean ± SD of 5 independent experiments. Figure S2: Knockout of CMP-sialic acid transporter SLC35A1 in AGA knockout HEK293T cells results in loss of immunostaining for the CMP-sialic acid transporter. Figure S3: Verification of the knockout of the sialic acid transporter SLC35A1 in AGA knockout HEK293T cells. Figure S4: Knockout of sialic acid transporter SLC35A1 gene in AGA knockout HEK293T cells improves AAV9/GFP transduction. Figure S5: Comparison of AAV9 transduction efficiency of HEK293T wildtype, SLC35A1/AGA double knockout, and SLC35A1 single knockout cells. Figure S6: Knockout of CMP-sialic acid transporter *SLC35A1* gene in HeLa cells and assessment of cell viability. (A) CRISPR/Cas9-mediated knockout of *SLC35A1* was confirmed by sequencing of PCRamplified genomic DNA surrounding the gRNA targe_ng site. Knockout cells show a frameshi muta_on (1 bp inser_on) in the vicinity of the Cas9 PAM sequence. (B) Cell viability of the parental and *SLC35A1* knockout HeLa cells was determined using the MTT assay and is given as rela_ve prolifera_on normalized to 24 h _me point of the parental cell line. The results represent the mean ± SD of 5 independent experiments.. Figure S7: Knockout of CMP-sialic acid transporter SLC35A1 gene in HeLa cells and assessment of cell viability. Figure S8: Knockout of SLC35A1 in HeLa cells improves AAV9 transduction. Figure S9: Graphs from Figure 6B,C with all data points.
