# Supplementary material for: Knockout of the CMP–Sialic Acid Transporter SLC35A1 in Human Cell Lines Increases Transduction Efficiency of Adeno-Associated Virus 9: Implications for Gene Therapy Potency Assays
Source: Cells. 2021 May 19;10(5):1259. doi: 10.3390/cells10051259 (PMC8160606; doi:10.3390/cells10051259)
Supplement: Supplementary file 1 [file cells-10-01259-s001.zip › cells-1213010-supplementary.pdf]

Suppl. Figure S1: *SLC35A1* knockouts

A

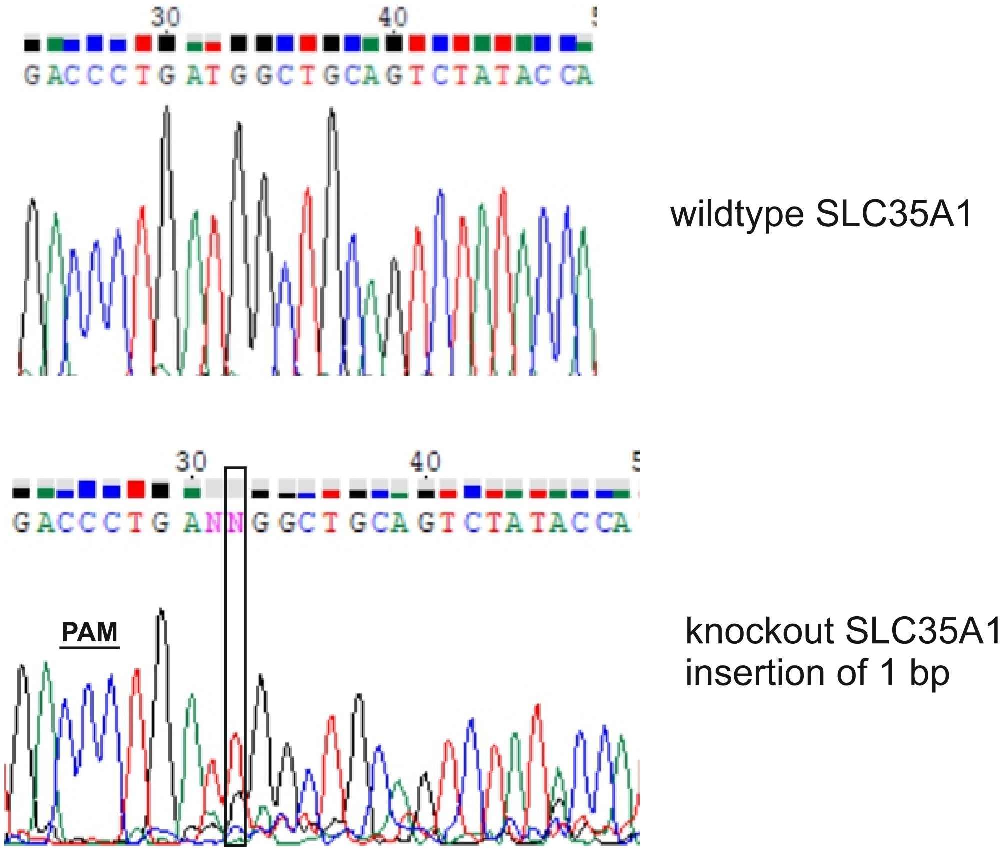

B

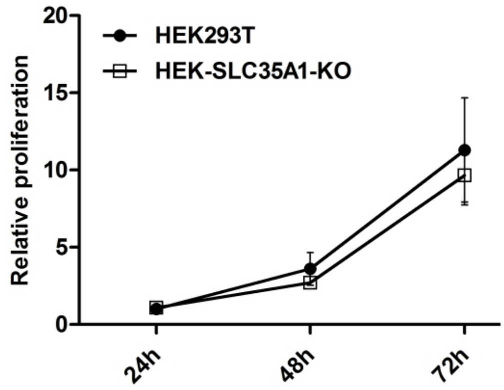

Suppl. Figure S1. Genomic sequencing and cell viability of *SLC35A1* knockout HEK293T cells. (A) CRISPR/Cas9-mediated knockout of the *SLC35A1* gene was confirmed by sequencing of PCR-amplified genomic DNA surrounding the gRNA targeting site. Knockout cells show frameshift mutations due to an insertion of 1 base 4 bp upstream of the Cas9 PAM sequence. (B) Cell viability of the parental and *SLC35A1* knockout cells was determined using the MTT assay and is given as relative proliferation normalized to 24 h time point of the parental cell line. The results represent the mean ± SD of 5 independent experiments.

Suppl. Figure S2: *AGA+SLC35A1* knockouts

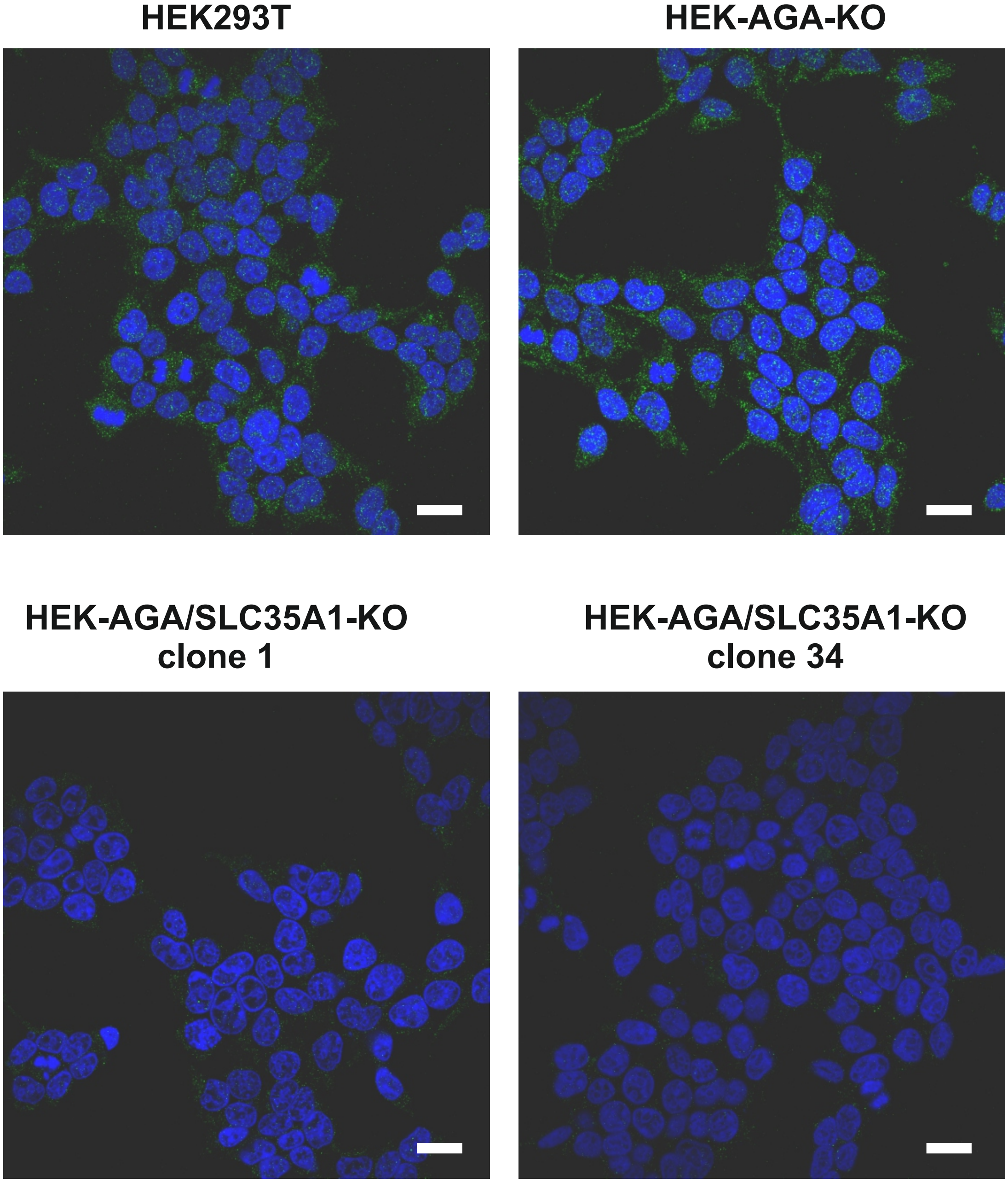

Suppl. Figure S2. Knockout of CMP-sialic acid transporter SLC35A1 in *AGA* knockout HEK293T cells results in loss of immunostaining for the CMP-sialic acid transporter. *SLC35A1* was knocked out via CRISPR/Cas9 in AGA-deficient HEK293T cells. HEK293T wildtype, *AGA* knockout and *SLC35A1/AGA* double knockout cells were grown on coverslips and stained for SLC35A1. Anti-rabbit IgG coupled to Alexa Fluor® 488 was used as a secondary antibody. In contrast to HEK293T wildtype and *AGA* knockout cells, *SLC35A1/AGA* double knockout cells lack specific signal for SLC35A1. Scale bar 20 µm.

Suppl. Figure S3: *AGA/SLC35A1* Knockouts

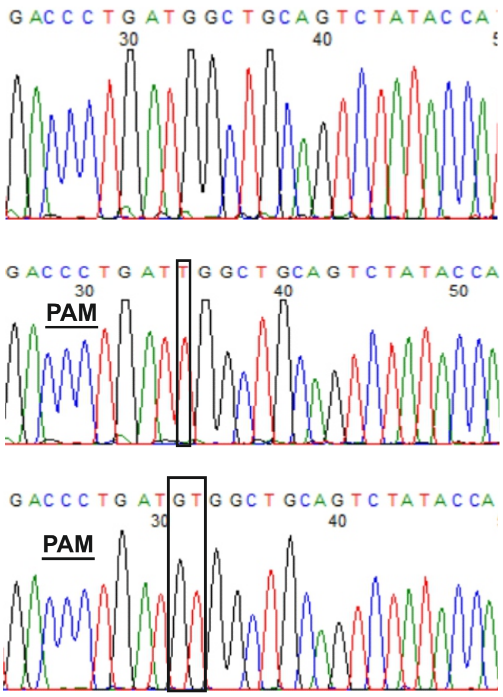

wildtype SLC35A1

knockout SLC35A1: clone#1
insertion of 1 bp

knockout SLC35A1: clone#34
insertion of 2 bp

Suppl. Figure S3. Verification of the knockout of the sialic acid transporter SLC35A1 in *AGA* knockout HEK293T cells. *SLC35A1* gene was knocked out via CRISPR/Cas9 in AGA-deficient HEK293T cells. Knockout of *SLC35A1* was confirmed in single cell clones by sequencing of PCR-amplified genomic DNA surrounding the gRNA targeting site. Two clones containing frame-shift mutations (insertion of 1 or 2 bp) in the vicinity of the Cas9 PAM sequence were chosen for further analysis.

Suppl. Figure S4: GFP fluorescence

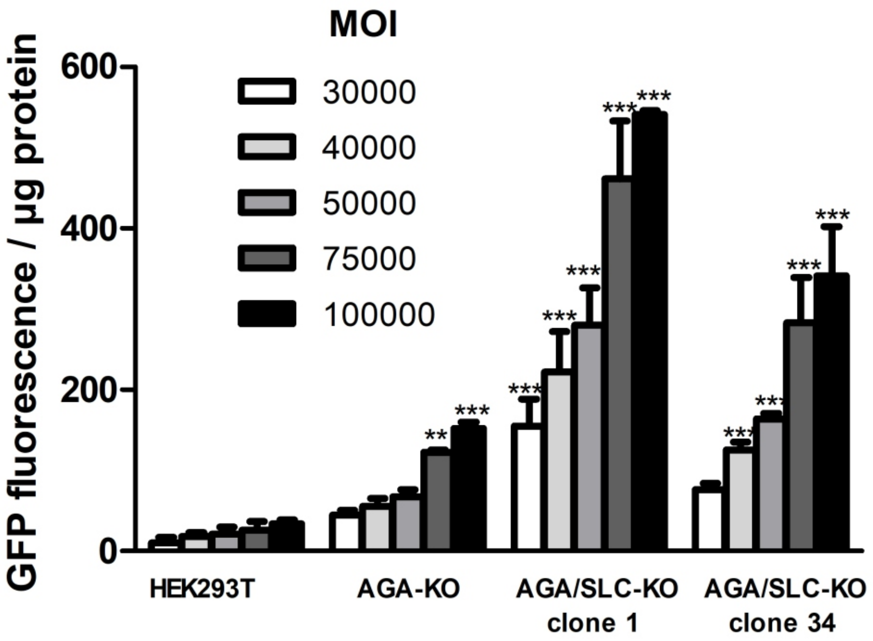

Suppl. Figure S4. Knockout of sialic acid transporter *SLC35A1* gene in *AGA* knockout HEK293T cells improves AAV9/GFP transduction. Cells were infected with increasing amounts of AAV9/GFP, and the GFP fluorescence was measured in cell lysates 72 h post transduction. Bars show the mean ± SD of 3 independent experiments, 2way ANOVA against HEK293T. ** $p \leq 0.01$, *** $p \leq 0.001$

Suppl. Figure S5: Number of GFP-positive cells

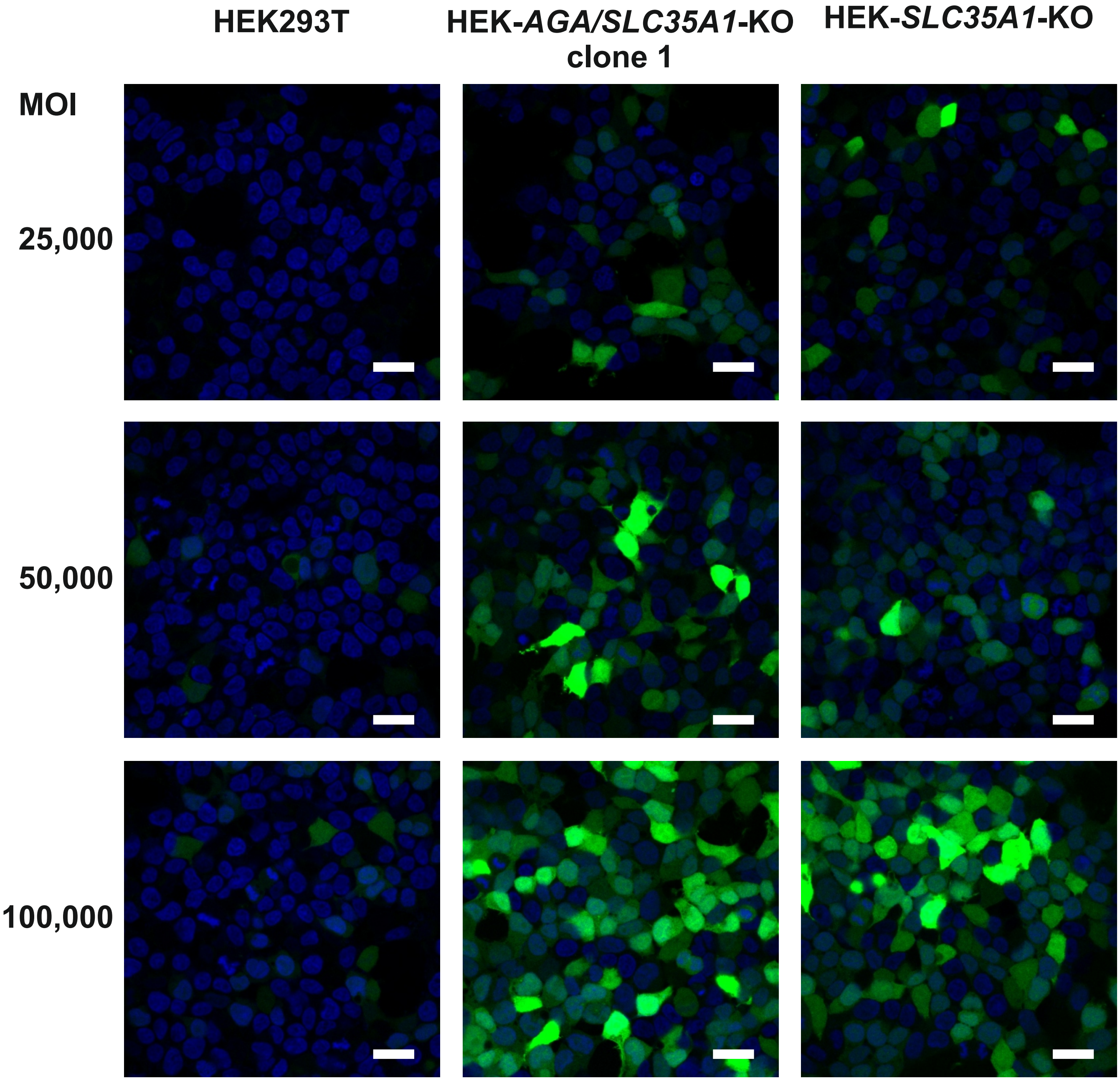

Suppl. Figure S5. Comparison of AAV9 transduction efficiency of HEK293T wildtype, *SLC35A1/AGA* double knockout, and *SLC35A1* single knockout cells. Cells were infected with increasing amounts of AAV9/GFP. 24 h post infection, the cells were transferred onto coverslips and grown for further 24 h. The cells were fixed and analyzed by fluorescence microscopy. Pictures are representative for 3 independent experiments. Scale bar 20 µm.

Suppl. Figure S6: Figures 3A - D with all data points

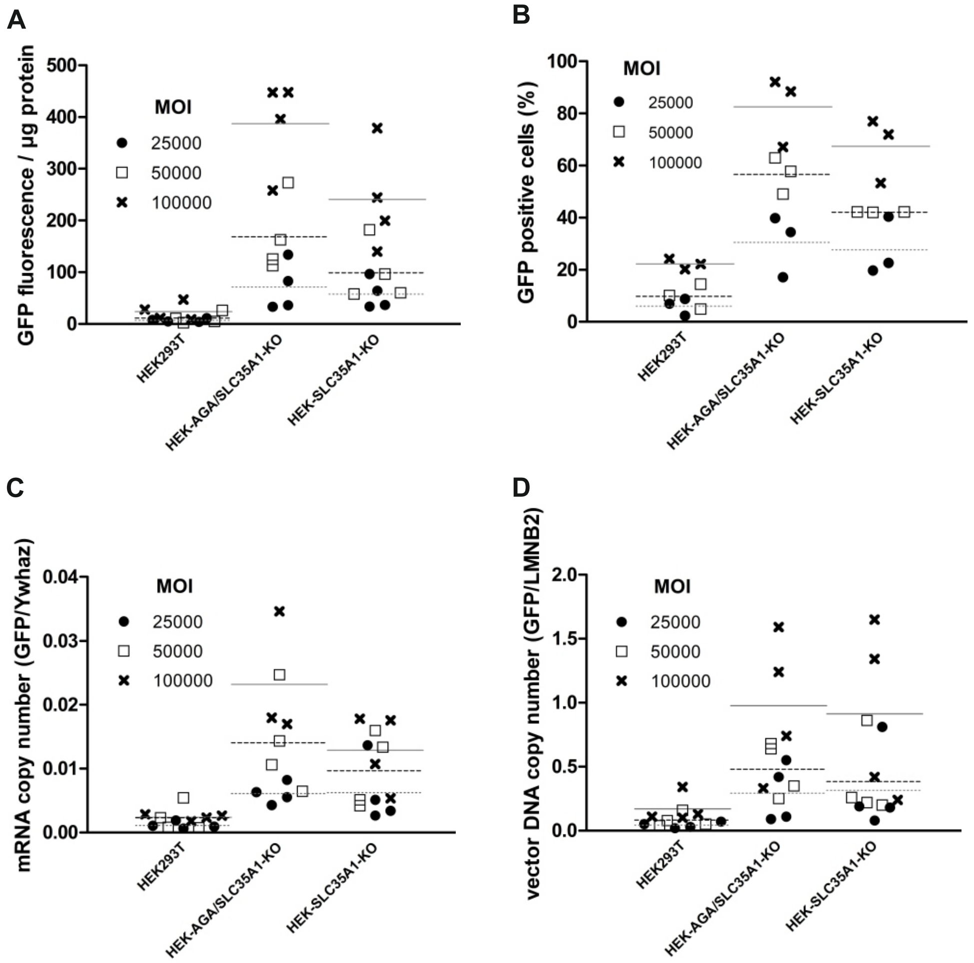

Suppl. Figure S6. Graphs from Figure 3 A-D with all data points. Comparison of AAV9/GFP transduction efficiency of HEK293T wildtype, *SLC35A1/AGA* double knockout and *SLC35A1* single knockout cells. Cells were infected with increasing amounts of AAV9/GFP. 72 h post-infection, the cells were harvested and (**A**) GFP fluorescence in cell lysates was quantified. (**B**) 24 h post-infection, cells were transferred onto coverslips and grown for another 24 h. The cells were fixed and analyzed by fluorescence microscopy. At least 300 cells per sample were counted per experiment. Only cells with a distinct green fluorescence signal were counted as GFP-positive. (**C-D**) Genomic DNA and mRNA were isolated from the cells and the copy number of the GFP mRNA (**C**) and vector DNA (**D**) were analyzed by quantitative real-time PCR. (**A-D**) Data are shown for 3 independent experiments. For clarity, the significances are shown only in the bar graphs in Figures 3A-D.

Suppl. Figure S7: HeLa *SLC35A1* knockout

A

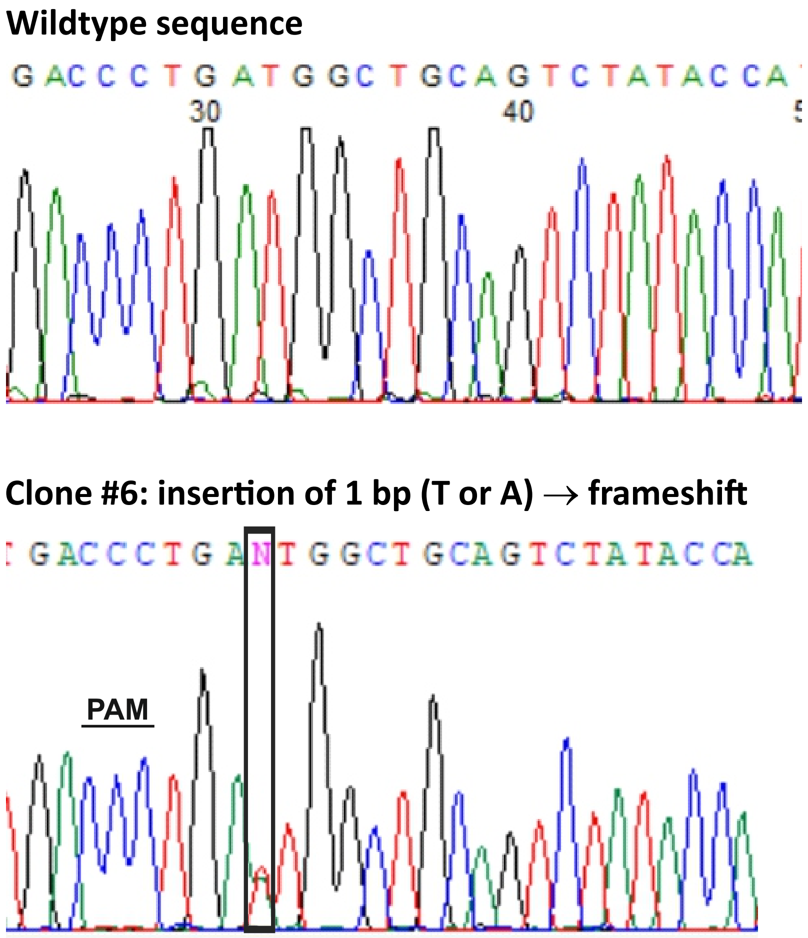

B

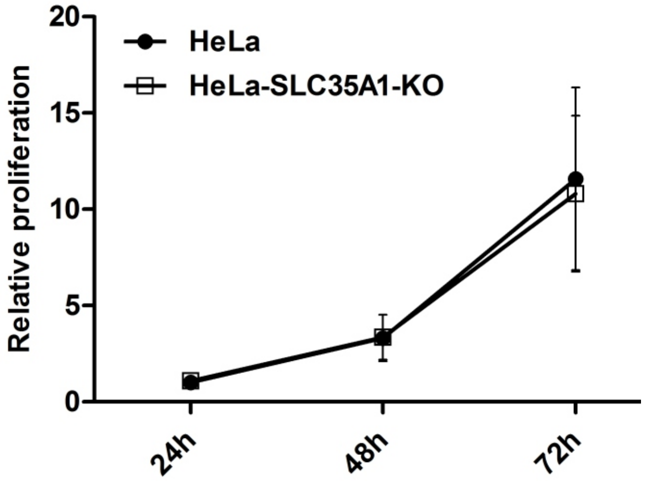

Suppl. Figure S7. Knockout of CMP-sialic acid transporter *SLC35A1* gene in HeLa cells and assessment of cell viability. (A) CRISPR/Cas9-mediated knockout of *SLC35A1* was confirmed by sequencing of PCR-amplified genomic DNA surrounding the gRNA targeting site. Knockout cells show a frameshift mutation (1 bp insertion) in the vicinity of the Cas9 PAM sequence. (B) Cell viability of the parental and *SLC35A1* knockout HeLa cells was determined using the MTT assay and is given as relative proliferation normalized to 24 h time point of the parental cell line. The results represent the mean ± SD of 5 independent experiments.

Suppl. Figure S8: GFP-positive HeLa *SLC35A1* knockout cells

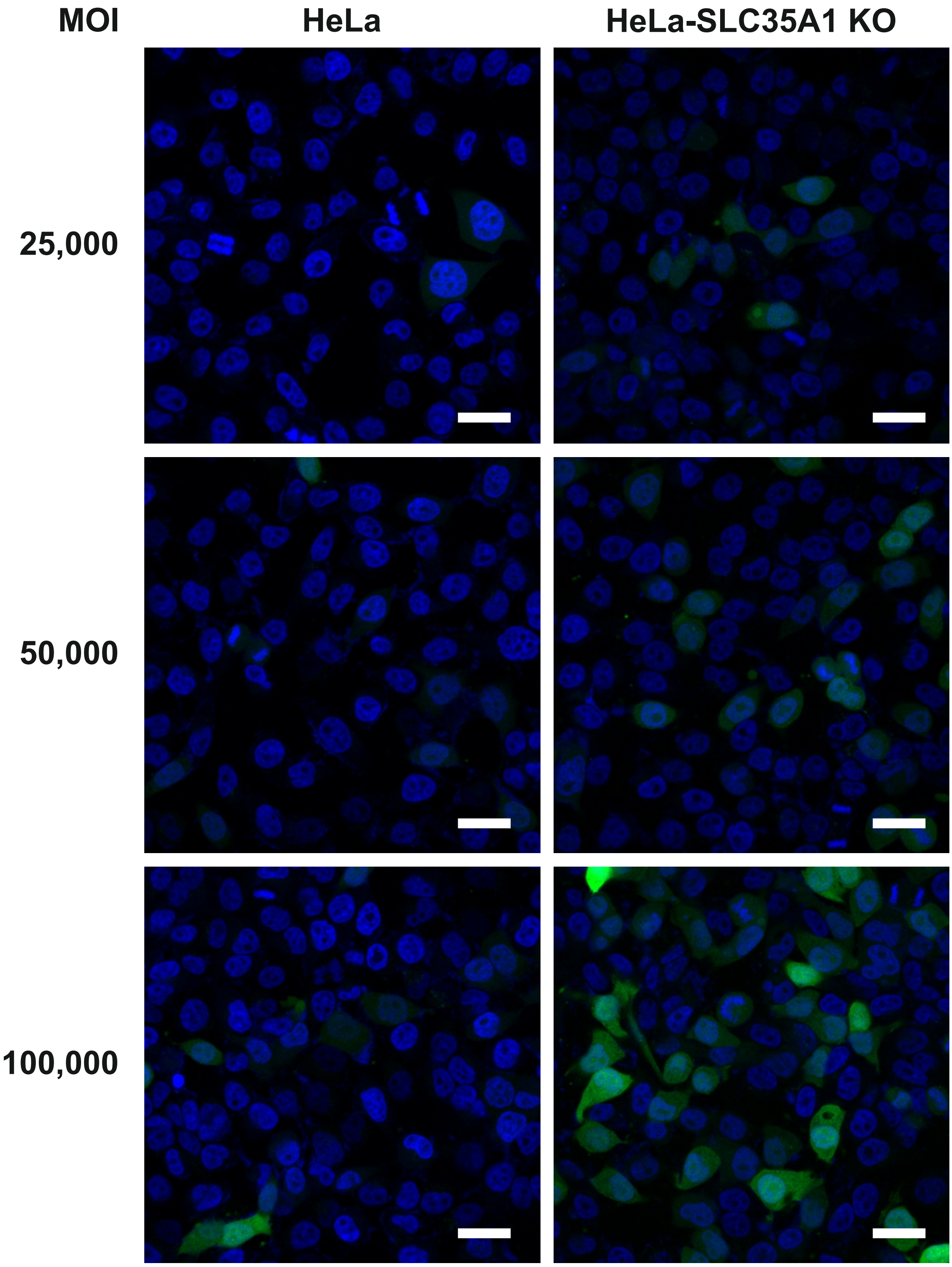

Suppl. Figure S8. Knockout of *SLC35A1* in HeLa cells improves AAV9 transduction. Cells were infected with increasing amounts of AAV9/GFP. 24 h past virus infection, cells were transferred onto coverslips and grown for another 24 h. Cells were fixed and analyzed by fluorescence microscopy. Pictures are representative for 3 independent experiments.

Suppl. Figure S9: Figure 6B and C with all data points

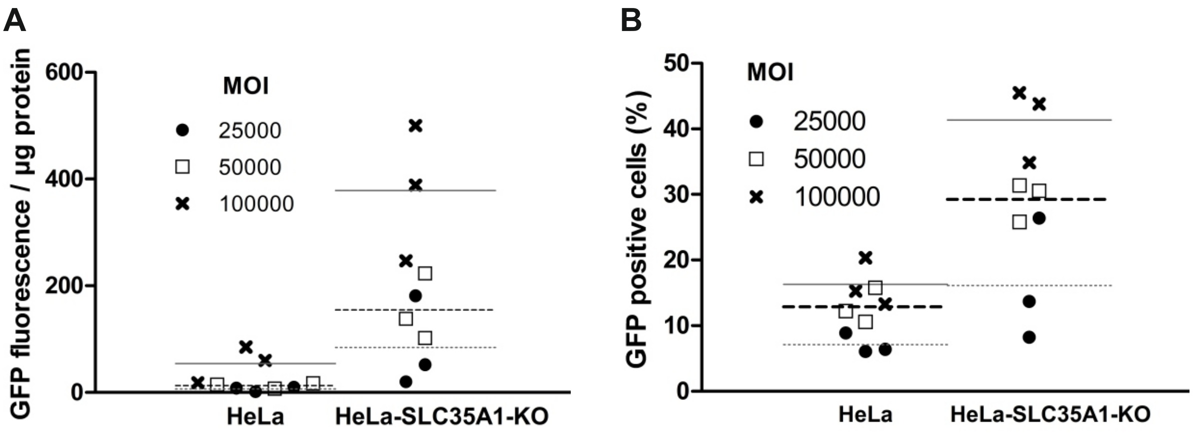

Suppl. Figure S9. Graphs from Figure 6B and C with all data points. (**A**) The cells were infected with increasing amounts of AAV9/GFP and the GFP fluorescence was measured in cell lysates 48 h post-transduction. (**B**) At 24 h post-infection, the cells were transferred onto coverslips and grown for further 24 h. The cells were fixed and analyzed by fluorescence microscopy. At least 300 cells per sample were counted per experiment. Only cells with a distinct green fluorescence signal were counted as GFP-positive.Data are shown for 3 independent experiments. For clarity, the significances are shown only in the bar graphs in Figures 6B and C.
